# Locoregional therapy of the primary tumour in *de novo* stage IV breast cancer in 216 066 patients: A meta-analysis

**DOI:** 10.1038/s41598-020-59908-1

**Published:** 2020-02-19

**Authors:** Ritika Gera, Hiba E. L. Hage Chehade, Umar Wazir, Salim Tayeh, Abdul Kasem, Kefah Mokbel

**Affiliations:** The London Breast Institute, Princess Grace Hospital, London, UK

**Keywords:** Breast cancer, Surgical oncology

## Abstract

Patients presenting with *de novo* stage IV metastatic breast cancer have a complex disease which is normally treated with palliative intent and systemic therapy. However, there is mounting evidence that resection of the primary tumour and/or localised radiotherapy (locoregional therapy; LRT) could be associated with overall survival improvements. We aimed to conduct a meta-analysis to inform decision making. Using the PubMed, Cochrane and Ovid SP databases, a literature review and meta-analysis were conducted to assess the effect of LRT on overall survival. Studies were analysed for the impact of LRT on survival. All forms of LRT resulted in a significant 31.8% reduction in mortality (N = 42; HR = 0.6823 (95% CI 0.6365; 0.7314)). Surgical resection resulted in a significant 36.2% reduction in mortality (N = 37; HR = 0.6379 (95% CI 0.5974; 0.6811)). The prospective trials reported a 19.23% reduction in mortality which was not statistically significant (N = 3, HR = 0.8077 (95% CI 0.5704; 1.1438). 216 066 patients were included. This is the largest meta-analysis regarding this question to date. Our meta-analysis shows that LRT of the primary tumour seems to improve overall survival in *de novo* stage IV disease. Therefore, this therapeutic option should be considered in selected patients after a careful multidisciplinary discussion.

## Introduction

The treatment of metastatic breast cancer remains a therapeutic challenge and systemic therapy currently represents the mainstay of treatment. For a significant proportion of stage IV patients, only palliative options may be feasible. Until recently there was no role for locoregional therapy (LRT) targeting the primary tumour outside of palliation and in the case of down-staged disease after neoadjuvant therapy^1^.

More recently, several studies have suggested a survival benefit for patients who underwent resection of the primary tumour in *de novo* stage IV disease^2,3^. Several mechanisms of action have been suggested, such as: tumour burden reduction^4^, cancer stem cell (CSC) elimination^5^, a reversal of tumour-induced immunosuppression^6^, a reduction in clonal heterogeneity^7^, disruption of self-seeding of the primary tumour^8^, disruption of the multidirectional movement of tumour cells between the primary tumour and distant sites^9,10^ and a reduction of tumour promoting activities mediated by CSCs^11–15^. Compared to the tumour bulk, CSCs are a small and dangerous subgroup of cells which are capable of self-renewal. They have unique characteristics which expose potential vulnerabilities to LRT. CSCs can differentiate and cause tumorigenesis^11^ and are strongly implicated in haematogenous and lymphatic metastasis^12^. There is evidence to suggest that CSCs localised in the primary tumour are likely to lend themselves to the development of metastatic disease^13–15^. Localised primary resection may therefore deplete the CSC pool and reduce distant metastases via disruption of the self-seeding mechanism.

Investigation of a potential role of LRT in stage IV breast disease is imperative if it could provide a means of improving the survival of patients with metastatic disease. Most evidence takes the form of retrospective studies using registry data, and there are significant contradictions in this literature. In order to better assess the literature surrounding this question, we performed a meta-analysis of the available studies pertaining to LRT in *de novo* stage IV breast cancer and its implications for patient survival. This is an update of our previously published meta-analysis^16^.

## Materials and Methods

### Data sources and searches

Complete searches of the PubMed, Ovid and Cochrane databases were undertaken to identify pertinent published literature. The key search terms differed depending on the database due to differentiating results. The PubMed search utilized the following search criteria: ((stage IV) OR (*de novo*)) AND (breast cancer) AND ((locoregional) OR (surgery) OR (radiotherapy)) from 2015/01/01 to 2019/10/31. The Ovid database search employed: AMED, Embase, and Ovid MEDLINE(R) and Epub Ahead of Print, In-Process and Other Non-Indexed Citations and Daily – without Revisions. These were searched thoroughly using the following terms: breast cancer and (*de novo* or primary metastatic or stage IV) and locoregional and (surgery or radiotherapy). Publications between 2015 and October 2019 were selected and subsequently analysed. Within the Cochrane database, the search criteria were: breast cancer and (*de novo* or primary metastatic or stage IV) and locoregional and (surgery or radiotherapy) with an applied date restriction during the period 2015–2019 as of October 2019. Studies reported in previous systematic reviews and meta-analyses were individually analysed for inclusion suitability. An assessment of the suitability of these articles was undertaken based on the inclusion and exclusion criteria listed below.

### Inclusion and exclusion criteria

Prospective clinical trials and retrospective studies were assessed. Abstracts were assessed for a description of the treatment modality used to treat stage IV breast cancer. If suitable, full texts were then examined. Reviews were excluded at the abstract stage, but their references were thoroughly searched for any relevant studies. The reference lists of full texts were also thoroughly screened for any relevant studies.

Studies must have included adult patients diagnosed with histologically confirmed stage IV breast cancer and distant metastases. The study must have reported overall survival outcomes and 95% confidence intervals (CIs) of patients who had undergone surgical resection, radiotherapy, or no treatment of the primary tumour. Conservative and extended resections were also considered. Studies were excluded from the meta-analysis if: there was a failure to report hazard ratios (HRs) and 95% CIs for overall survival, the full text was not available for data extraction, all patients received surgical resection of the primary tumour, patients received surgical resection of visceral metastases, and they were reviews/case reports/letters/commentaries.

### Data extraction and management

Data was extracted by authors independently. The following characteristics were recorded: population characteristics, the number of patients undergoing locoregional surgery and/or locoregional radiotherapy, the number of patients not undergoing any locoregional treatment, the HRs and 95% confidence intervals of overall survival from each of these groups, the presence of bone or any other metastases, the receptor statuses of estrogen, progesterone and HER2, the number of distant metastatic sites, the number of patients undergoing systemic therapy, the performance status, tumour and nodal burden, authors, publication dates, confounding factors/evidence of sample selection bias, bias in measurement of exposures and outcomes, selective reporting of outcomes and analyses, study designs and statistical analyses.

### Measures of treatment effects and statistical analysis

As a function of primary breast cancer surgery and/or radiotherapy with or without other treatment modalities, HRs and confidence intervals were retrieved for each study. Reduced risk of mortality was deduced by HR < 1 when comparing patients treated with LRT versus patients not treated with LRT.

Fixed and random effects models were used to perform a meta-analysis of HRs for calculating a single pooled effect of LRT on stage IV disease. For risk of bias analysis, the following tests were conducted in addition to creating a funnel plot: Duval and Tweedie’s trim and fill, Classic fail-safe N, Begg and Mazumdar Rank Correlation Test of funnel plot asymmetry, Egger’s Test for funnel plot asymmetry. For sensitivity analysis, the random effects model was re-estimated with different approaches to estimation of between-study variance tau. Furthermore, all estimates were performed with and without application of the Hartung-Knupp method. Finally, pooled estimates for LRT (the results for surgery only, radiotherapy only, or surgery and radiotherapy combined), surgery alone, and prospective studies alone were presented, which is an analysis of sensitivity. Among the included studies, the following tests were used to assess and quantify statistical heterogeneity: Cochran’s Q test, χ^2^ test, and I^2^ statistic. A classical forest plot was used to report the results of the meta-analyses.

## Results

### Literature search results and characteristics of the included studies

**Figure 1:**
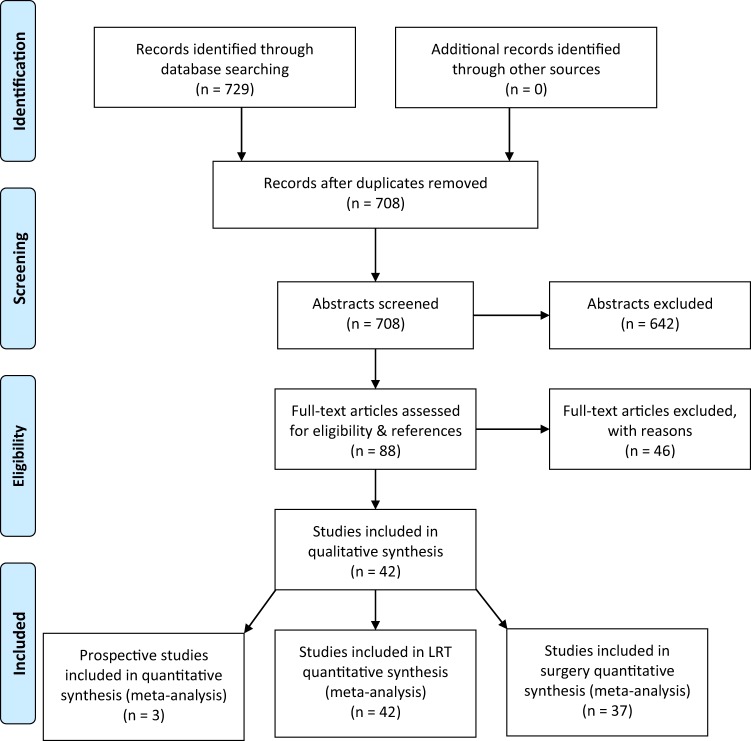
A PRISMA flowchart summarising the selection process for publications included in the meta-analysis.

A total of 729 studies were initially assessed (585 from PubMed, 114 from Ovid, and 30 from the Cochrane Library). 708 abstracts were left after 21 duplicates were removed. Then, 642 abstracts were excluded at initial screening, leaving 88 full-text articles for assessing eligibility and references. Finally, 46 full texts were excluded after examination of the full text. More information about the excluded studies, alongside explanations for their exclusion, can be found in Supplementary Table 1. 42 studies^17–58^ met the criteria for analysing the efficacy of all LRT (surgery only, radiotherapy only, or surgery and radiotherapy combined). 37 studies^17–21,23–29,31,33,34,36–54,56–58^ studies were suitable for the analysis of surgery-only treatment of the primary. Finally, the three prospective studies were analysed in a separate meta-analysis^18,20,42^. In total, this meta-analysis therefore included 216 066 patients reported between 2002 and 2019. These results are summarised in the PRISMA flowchart outlined in Fig. 1. A table outlining information on populations, interventions, controls, outcomes, designs, and statistical analyses from all included studies can be found in Supplementary Table 2.

### All LRT

**Figure 2:**
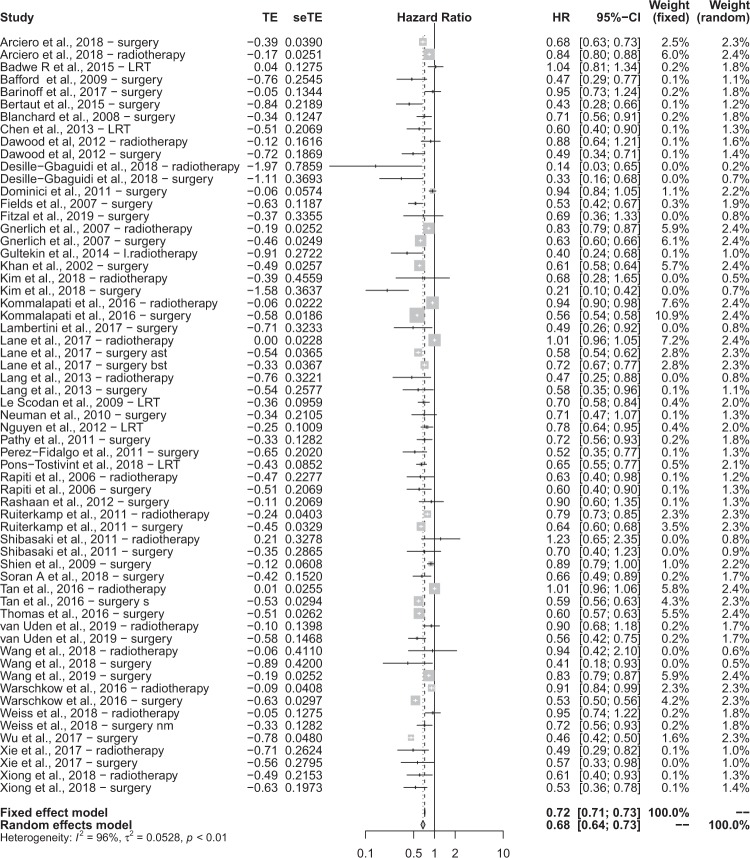
A forest plot summarising the results of the studies included in ‘LRT’.

A total of 61 data inputs from 42 studies were retrieved, which have been reported as a forest plot in Fig. 2. Firstly, we evaluated the null hypothesis, which specified that all treatment effects were equal to zero and all HRs equate to 1. The HR was calculated by the random effects model on the combined effect of locoregional surgery and radiotherapy versus no treatment. Highly significant heterogeneity was observed in both the χ^2^ and I^2^ tests, hence the implication that all studies evaluate the same effect was decisively rejected. A random effects model was used to calculate the HR to account for variability between and within studies.

**Figure 3:**
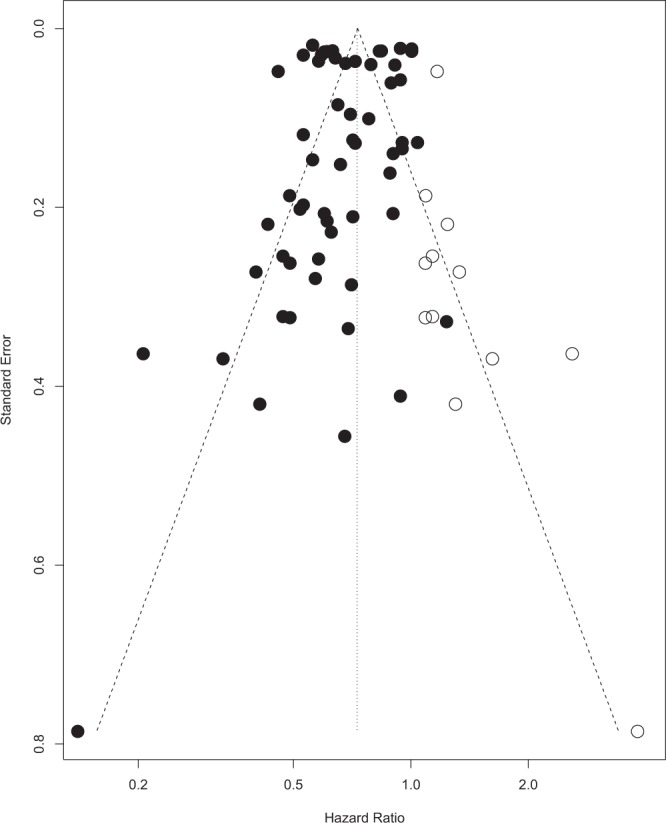
A funnel plot used for statistical analysis of the studies included in ‘LRT’.

HR = 0.6823 (95% CI 0.6365; 0.7314), hence a statistically significant 31.77% risk reduction was observed in patients receiving LRT compared to no treatment. To assess for bias, a funnel plot with Duval and Tweedie’s trim and fill method was created, shown in Fig. 3. The scatterplot nature of the studies indicates some positive relation between study size and its reported effect. There are a relatively large number of studies outside the 95% CI, indicating significant heterogeneity between reported results. Duval and Tweedie’s trim and fill suggests that 8 studies should be imputed to correct for asymmetry, which are represented on the funnel plot using unpainted dots. After adjustment using Duval and Tweedie’s trim and fill, the following significant results are reported: HR = 0.7281 (95% CI 0.6805; 0.7791). Nevertheless, tests using Begg and Mazumdar Rank Correlation and Egger’s calculations do not provide evidence of asymmetry (Begg and Mazumdar Rank Correlation Test, P = 0.2065; Egger’s test, P = 0.4015).

Although there is evidence of publication bias, (Q statistic = 1387.48 and I^2^ statistic = 95.7% [95.0%; 96.3%]), the Classic fail-safe N test (Rosenberg method) suggests that as many as 44 425 studies would be required to reduce the significance level of the pooled effect size to 5%. This supports the conclusion that the effect sizes are significant, even in view of some possible publication bias.

### Surgery only

**Figure 4:**
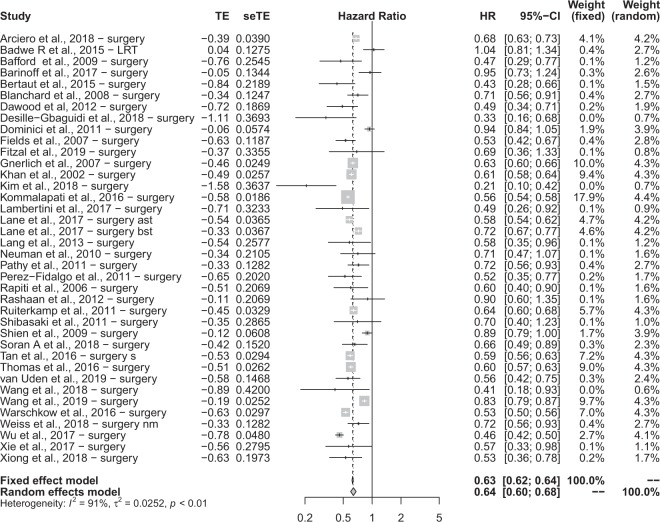
A forest plot summarising the results of the studies included in ‘surgery’.

A total of 38 data inputs from 37 studies were retrieved, which have been reported as a forest plot in Fig. 4. Firstly, we evaluated the null hypothesis, which specified that all treatment effects were equal to zero and all HRs equate to 1. The HR was calculated by the random effects model on the combined effect of locoregional surgery and radiotherapy versus no treatment. Highly significant heterogeneity was observed in both the χ^2^ and I^2^ tests, hence the implication that all studies evaluate the same effect was decisively rejected.

**Figure 5:**
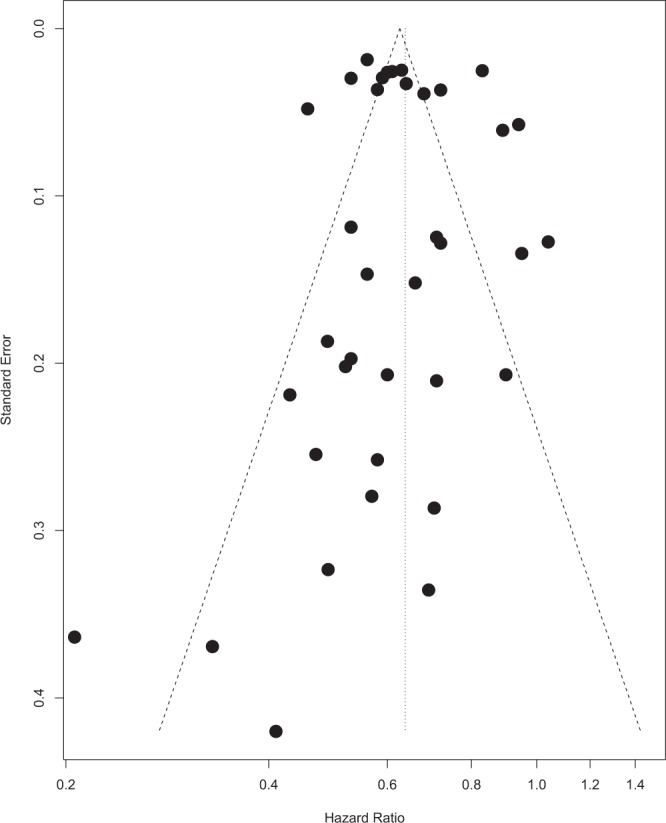
A funnel plot used for statistical analysis of the studies included in ‘surgery’.

A random effects model was used to calculate the HR to account for variability between and within studies. HR = 0.6379 (95% CI 0.5974; 0.6811), hence a statistically significant 36.21% risk reduction was observed in patients receiving surgery compared to no treatment. To assess for bias, a funnel plot with Duval and Tweedie’s trim and fill method was created, shown in Fig. 5. The scatterplot nature of the studies indicates some positive relation between study size and its reported effect. There are no studies outside the 95% CI, indicating no significant heterogeneity between reported results. Furthermore, tests using Begg and Mazumdar Rank Correlation and Egger’s calculations do not provide evidence of asymmetry (Begg and Mazumdar Rank Correlation Test, P = 0.4659; Egger’s test, 0.7807).

Although there is some evidence of publication bias, (Q statistic = 407.71and I^2^ statistic = 90.9% [88.5%; 92.8%]), the Classic fail-safe N test (Rosenberg method) suggests that as many as 34952 studies would be required to reduce the significance level of the pooled effect size to 5%. This supports the conclusion that the effect sizes are significant, even in view of some possible publication bias.

### Prospective studies

**Figure 6:**
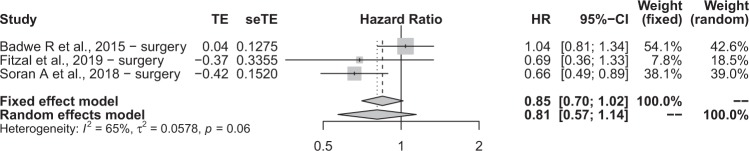
A forest plot summarising the results of the studies included in ‘3 prospective studies’.

A total of 3 data inputs from 3 studies were retrieved and reported as a forest plot in Fig. 6. Firstly, we evaluated the null hypothesis, which specified that all treatment effects were equal to zero and all HRs equate to 1. The HR was calculated by the random effects model on the combined effect of locoregional surgery and radiotherapy versus no treatment. Highly significant heterogeneity was observed in both the χ^2^ and I^2^ tests, hence the implication that all studies evaluate the same effect was decisively rejected.

**Figure 7:**
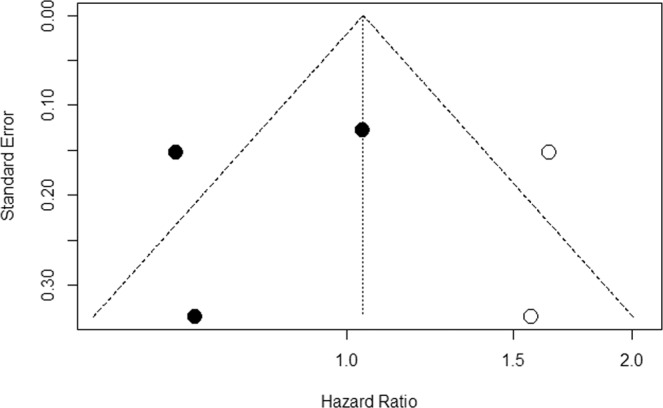
A funnel plot used for statistical analysis of the studies included in ‘3 prospective studies’.

A random effects model was used to calculate the HR to account for variability between and within studies. HR = 0.8077 (95% CI 0.5704; 1.1438) and this suggests a 19.23% risk reduction in patients receiving surgery compared to no treatment that did not reach statistical significance. To assess for bias, a funnel plot with Duval and Tweedie’s trim and fill method was created, shown in Fig. 7. The scatterplot nature of the studies indicates some positive relation between study size and its reported effect. There is 1 study outside the 95% CI, indicating heterogeneity between reported results. After adjustment using Duval and Tweedie’s trim and fill, the following results are reported: HR = 1.04 (95% CI 0.7102; 1.5228). Furthermore, there is some evidence of publication bias, (Q statistic = 5.65 and I^2^ statistic = 64.6% [0.0%; 89.9%].

### Significant prognostic factors, incidence in patient cohorts, and associated overall survival

The following information was retrieved from all papers examined in this meta-analysis and assessed for significance in receipt of LRT: bony metastasis, HER2 positivity, ER/PR-positivity, number of metastatic sites, and performance status. Investigators examined whether there was a significant difference in prognostic factors between patients who received LRT versus no LRT. Information regarding the distribution of populations according to these significant factors can be found as Supplementary Table 3.

The ten largest retrospective studies were assessed for the impact on overall survival of prognostic factors when LRT was applied^17,21,23,25,27,43,44,46,48,50^. The three prospective studies^18,20,42^ were all extremely heterogeneous in their findings and are expanded upon further in the discussion.

Khan *et al*. found that most patients had a total mastectomy, but identical benefit to overall survival could have been achieved with partial mastectomy accompanied by clear margins. Furthermore, partial and total mastectomy both offered significant survival benefit compared to no surgery. Tumour size was a weak predictor of survival and the extent of nodal involvement did not predict survival.

Kommalapati *et al*. observed significant survival benefit with the presence of clear margins and recorded no significant difference in overall survival between breast conserving surgery and mastectomy. Patients with lympho-vascular invasion had significantly worse overall survival. This group developed a prognostic scoring model where they sorted patients with according to severity of disease characteristics. Interestingly, patients with worse prognostic scoring had decreased overall survival when they underwent LRT compared to the control patient cohort, suggesting that LRT should be avoided in patients with worse prognosis.

Arciero *et al*. found that there was increased overall survival in surgical patients with hormone-receptor positive tumours, and lack of radiation therapy or the presence of positive margins were associated with worsened overall survival. They also observed that patients who received surgery and systemic therapy had the longest median overall survival, but multivariable and propensity-matched analyses showed persistent improvement when surgical intervention occurred, with or without systemic therapy.

Lane *et al*. noted that the greatest survival benefit was seen in ER-positive patients who underwent surgery following systemic therapy. Nevertheless, both groups had significantly better overall survival than patients who had systemic therapy alone. Although Lane *et al*. noted some concerns regarding selection bias, their observations were considered significant; patients who had surgery prior to systemic therapy had poorer overall survival despite generally having smaller tumours with lesser nodal involvement.

Wang *et al*. found that patients who underwent LRT with single organ/bone and additional visceral or non-visceral metastases were conferred significant survival benefit. They also observed surgical benefit in patients with bone and additional visceral/non-visceral metastases. Moderate tumour burden had no significant difference on survival. However, Wang *et al*. also concluded that patients with distant metastases occurring simultaneously in the liver and lung experienced significantly increased likelihood of death when primary resection occurred.

Interestingly, Wu *et al*. concluded that surgery improved overall survival regardless of breast cancer subtype but according to multivariate analysis, moderately/poorly differentiated tumours which were larger than 5 cm had significantly worse survival outcomes. Patients with well-differentiated, small tumours with lower nodal burden experienced significant survival benefit across all breast cancer subtypes.

Concurrently, Tan *et al*. observed significant overall survival benefits with: T0–T4 cancers, all sites of (single or multiple) metastases, patients with/without radiation treatment and patients with hormone receptor positive/negative disease when LRT was administered. Tan *et al*. also noted that primary resection combined with metastatic resection offered the best survival benefit, but in isolation, primary resection was superior to metastatic resection. Interestingly, in hormone receptor negative patients, the survival benefit observed with joint primary and metastatic resection was insignificant compared to primary resection alone.

Thomas *et al*. concluded that all patients, irrespective of tumour size, significantly benefitted from surgery. Furthermore, hormone receptor-positive and negative patients achieved significant survival benefit from surgery. Following surgery, women with tumours which measured 2 cm or smaller saw an additional survival improvement of 11 months.

Warschkow *et al*. and Gnerlich *et al*. did not provide any further information on the effect of LRT on prognostic factor-specific survival.

## Discussion

The result of our meta-analysis of the literature regarding the role of LRT in Stage IV disease suggests that it could reduce mortality in such patients by 31.8%. Surgery would specifically reduce mortality by 36.2%. These findings significantly strengthen the argument for curative resection and radiotherapy of the primary tumour in metastatic breast cancer. However, prospective studies show a mortality reduction of 19.2% with locoregional intervention compared to no locoregional intervention, which unveils a significant disparity between the published retrospective and prospective data and merits further investigation.

Traditionally, it was believed that once a cancer metastasises, there is no therapeutic justification for resection of the primary tumour. However, as discussed above, there is mounting evidence that the primary tumour continues to play a role in metastatic disease, which would account for the benefits ascribed to LRT.

There are several theories forwarded to account for this. The most plausible model is that the removal of the primary tumour reduces the tumour burden, thereby increasing response to systemic therapy. With reduced cancerous tissue, a complete response to therapy is more likely^9^. Another factor is that the primary tumour is that the main source of new clonal lines of cancer cells, which are implicated in the emergence of resistance to therapy and the appearance of more aggressive disease phenotypes. LRT would disrupt this process^15^.

Other, less attested, hypotheses include the role of cancer stem cells in the primary tumour. This hypothesis ascribes these findings to the removal the purported cancer stem cells in the primary tumour, which are believed to maintain the microenvironments of the tumour, which recruit other cells into the pathology and propagate the disease^59^. Another suggestion is that removal of the primary tumour reverses tumour-induced immunosuppression. This model is better described in lymphomas but is increasingly being explored in breast cancer^14^. It is suggested that the primary tumour encourages metastasis by induction of systemic inflammation via IL-1B, which leads to the expression of IL-17 from γδ T cells. This results in tumour-induced neutrophils which would suppress cytotoxic CD8+ T cell effector function. The weakening of the immune response to the tumour cells enables distant metastases^60^. In animal models, surgical resection of the primary tumour led to neutrophil depletion. However, decreased multi-organ metastasis occurred only in the early phase and had little impact on late phase disease progression^61^. This latter observation may suggest that to be of benefit, LRT in metastatic breast disease should be attempted early, and may not be as efficacious in patients with more extensive distant disease.

Finally, it has been suggested that removal of the primary tumour disrupts the process of tumour self-seeding. In this process, circulating cells from the primary tumour seed the distant metastases, which in turn seeds the primary. This is theorised to encourage the emergence of more aggressive forms of the disease with aggressive spread of metastases^16^.

Whilst most of the studies demonstrated a benefit in performing LRT for the primary in metastatic breast disease, some studies contradicted this finding. A notable example is the prospective trial by Badwe *et al*., in a randomised controlled trial (RCT) comparing post-primary systemic therapy in patients who received LRT or did not, which reported an increase in distant metastases after LRT, along with decreased locoregional progression. An explanation offered by the authors was that the primary tumour surgery may have led to tumour cell shedding. Badwe *et al*. reported no population groups which enjoyed significant improvement in survival after receiving LRT. However, the study has flaws in that some subgroups were significantly undertreated^18^.

Fitzal *et al*. also failed to find a survival benefit for surgery and luminal A patients had significantly worse survival following surgery. However, the study was marred by poor recruitment and was prematurely concluded, with only 90 participants^62^. Fitzal *et al*. argued that the presence of stem cells in sites of distant metastases may have led to the lack of clear benefit for LRT of the primary tumour. Furthermore, some publications have speculated that surgery to the primary tumour could trigger metastases by inducing cancer cell shedding into the circulation^63^. Other authors hypothesise that primary tumours systematically inhibit the progress of metastasis initiating cells by preventing their proliferation and differentiation in distant metastatic sites via interleukin-1B. This is said to be independent from the adaptive immune response^64^.

In direct contrast, Soran *et al*. conducted a RCT which found a significant survival benefit for the LRT group compared to the systemic therapy group (HR = 0.66; 95%CI 0.49–0.88). Patients with HER2-negative, HR-positive, or solitary bone-only metastasis experienced significantly greater survival after receiving LRT. There was no significant difference in survival three years following intervention but there was a significant divergence in survival at five years.

In view of the countervailing arguments regarding this question, this meta-analysis serves as a systematic and robust way of interpreting the literature. At the time of writing, this is the largest meta-analysis regarding this question, and strengthens the case for increased use of LRT in metastatic disease.

This publication has followed and met AMSTAR-2 criteria. The research questions included PICO, which have been further expanded upon by creating a summary table describing the key characteristics of each included study. Due to the paucity of randomised trials exploring LRT use in stage IV disease, this was a meta-analysis which included retrospective and prospective data; both sets provide a complete picture of the effect. However, withstanding for retrospective bias, separate analyses was conducted to specifically examine the prospective trials. Throughout the course of the literature review, a wide range of databases were examined, published reviews were checked, and reference lists were reviewed. A list of excluded studies, with justifications, has been provided. Confounding, sample selection bias, bias in measurement of exposures and outcomes, and selective reporting were all assessed as factors within individual studies which may contribute to bias; this information can be found in Supplementary Table 4. The meta-analysis was also examined for heterogeneity and publication bias.

However, our meta-analysis has some limitations. Data sets from publications specifically examining HER2-positive metastatic disease could not be separated from other pathology. Studies varied greatly in terms of treatment protocols and duration of follow-up, and we used data from multivariate and univariate analyses (depending on availability). The incidence of positive resection margins differed between studies and were not reported uniformly, hence we could not assess its effect on overall survival. However, it should be noted that margin negativity tended towards significantly improved overall survival. Finally, an inherent selection bias of patients should be acknowledged. This was noted by Cady *et al*.^65^, who believed that case selection bias could explain the perceived survival advantage observed in the LRT group.

Such concerns highlight the need for more prospective studies regarding the use of LRT in metastatic disease. This current study is an increment upon our previous meta-analysis^16^, and represents a gradual accumulation of evidence in favour of more powerful prospective studies in the future, which would hopefully inform clinical guidelines regarding this question. The question of treatment modalities for this segment of the population is important because they continue to face poor prognoses and need a therapeutic breakthrough.

### Concluding remarks

It appears from several retrospective individual studies that patients with metastases limited to bone^35,46^, HER2-positive disease^31,43,54,55^, favourable response to primary systemic therapy, and a resectable primary tumour are most likely to derive significant survival benefit from LRT. Conflictingly, surgery did not improve overall survival in some patients with bone/soft tissue metastases^41^, and one prospective trial showed a greater survival benefit for HER2-negative patients^42^, hence more prospective information is required. Prospective trials also indicated favourable outcomes for patients with a positive response to systemic therapy and good primary resectable margins.

Further research is required to establish the biochemical basis by which primary tumours influence the location, development and growth of metastatic tumours. This information should aid in the identification of patients who would most benefit from LRT. It has been suggested that CTCs can be used to stage metastatic disease and characterise it as indolent or aggressive^66^; this could be a novel mechanism to identify patients with indolent disease and who will most likely to benefit from primary LRT.

Stage IV breast cancer survival continues to improve due to the emergence of novel targeted therapies. Therefore, LRT of the primary tumour may achieve additional survival benefits in patients who respond well to initial primary systemic therapy, especially with HER2- and/or ER-positive disease confined to the bone. Patients with extensive visceral metastases and/or triple negative disease with poor systemic response are unlikely to derive a survival benefit from LRT of the primary tumour, but LRT has the potential to achieve a palliative role.

## Supplementary information

Supplementary Dataset 1.

Supplementary Dataset 2.

Supplementary Dataset 3.

Supplementary Dataset 4.

## Data Availability

The datasets used and/or analysed during the current study are available from the corresponding author on reasonable request. The datasets generated during and/or analysed during the current study are available from the corresponding author on reasonable request.

## References

[1] Senkus E (2015). Primary breast cancer: ESMO Clinical Practice Guidelines for diagnosis, treatment and follow-up. Ann. Oncol..

[2] Gulack BC (2016). Surgical Resection of the Primary Tumor in Stage IV Colorectal Cancer Without Metastasectomy Is Associated With Improved Overall Survival Compared With Chemotherapy/Radiation Therapy Alone. Dis. Colon Rectum.

[3] Sokolov, M. Surgical approach in locally advanced colorectal cancer–combined, extended and compound surgery. *Khirurgiia (Sofiia)*. 29–50 (2013). 24800318

[4] Rashid OM (2013). Resection of the primary tumor improves survival in metastatic breast cancer by reducing overall tumor burden. Surg. (United States).

[5] Karnoub AE (2007). Mesenchymal stem cells within tumour stroma promote breast cancer metastasis. Nature.

[6] Danna EA (2004). Surgical Removal of Primary Tumor Reverses Tumor-Induced Immunosuppression Despite the Presence of Metastatic Disease. Cancer Res..

[7] Cummings DM, Snyder JS, Brewer M, Cameron HA, Belluscio L (2014). Adult Neurogenesis Is Necessary to Refine and Maintain Circuit Specificity. J. Neurosci..

[8] Norton L, Massagué J (2006). Is cancer a disease of self-seeding?. Nature Medicine.

[9] Kim M-Y (2009). Tumor Self-Seeding by Circulating Cancer Cells. Cell.

[10] Comen E, Norton L, Massagué J (2011). Clinical implications of cancer self-seeding. Nat. Rev. Clin. Oncol..

[11] Yu Z, Pestell TG, Lisanti MP, Pestell RG (2012). Cancer stem cells. Int. J. Biochem. Cell Biol..

[12] LI S, LI Q (2014). Cancer stem cells and tumor metastasis. Int. J. Oncol..

[13] Abraham BK (2005). Prevalence of CD44+/CD24−/low cells in breast cancer may not be associated with clinical outcome but may favor distant metastasis. Clin. Cancer Res..

[14] Liu H (2010). Cancer stem cells from human breast tumors are involved in spontaneous metastases in orthotopic mouse models. Proc. Natl. Acad. Sci..

[15] Balic M (2006). Most Early Disseminated Cancer Cells Detected in Bone Marrow of Breast Cancer Patients Have a Putative Breast Cancer Stem Cell Phenotype. Clin. Cancer Res..

[16] Headon H, Wazir U, Kasem A, Mokbel K (2016). Surgical treatment of the primary tumour improves the overall survival in patients with metastatic breast cancer: A systematic review and meta-analysis. Mol. Clin. Oncol..

[17] Arciero Cletus, Liu Yuan, Gillespie Theresa, Subhedar Preeti (2019). Surgery and survival in patients with stage IV breast cancer. The Breast Journal.

[18] Badwe R (2015). Locoregional treatment versus no treatment of the primary tumour in metastatic breast cancer: an open-label randomised controlled trial. Lancet Oncol..

[19] Fields RC (2007). Surgical Resection of the Primary Tumor is Associated with Increased Long-Term Survival in Patients with Stage IV Breast Cancer after Controlling for Site of Metastasis. Ann. Surg. Oncol..

[20] Fitzal F (2019). Impact of Breast Surgery in Primary Metastasized Breast Cancer. Ann. Surg..

[21] Gnerlich J (2007). Surgical Removal of the Primary Tumor Increases Overall Survival in Patients With Metastatic Breast Cancer: Analysis of the 1988–2003 SEER Data. Ann. Surg. Oncol..

[22] Gultekin M (2014). Impact of locoregional treatment on survival in patients presented with metastatic breast carcinoma. Breast.

[23] Khan, S. A., Stewart, A. K. & Morrow, M. Does aggressive local therapy improve survival in metastatic breast cancer? *Surgery* **132**, 620–6; discussion 626-7 (2002). 10.1067/msy.2002.127544 12407345

[24] Kim HJ (2018). Survival Benefit of Surgical Removal of Primary Tumor in Patients With Stage IV Breast Cancer. Clin. Breast Cancer.

[25] Kommalapati A (2018). A prognostic scoring model for survival after locoregional therapy in *de novo* stage IV breast cancer. Breast Cancer Res. Treat..

[26] Lambertini M (2017). Patterns of Care and Clinical Outcomes of HER2-positive Metastatic Breast Cancer Patients With Newly Diagnosed Stage IV or Recurrent Disease Undergoing First-line Trastuzumab-based Therapy: A Multicenter Retrospective Cohort Study. Clin. Breast Cancer.

[27] Lane WO (2019). Surgical Resection of the Primary Tumor in Women With *De Novo* Stage IV Breast Cancer. Ann. Surg..

[28] Lang JE (2013). Primary Tumor Extirpation in Breast Cancer Patients Who Present with Stage IV Disease is Associated with Improved Survival. Ann. Surg. Oncol..

[29] Bafford AC (2009). Breast surgery in stage IV breast cancer: impact of staging and patient selection on overall survival. Breast Cancer Res. Treat..

[30] Le Scodan R (2009). Breast Cancer With Synchronous Metastases: Survival Impact of Exclusive Locoregional Radiotherapy. J. Clin. Oncol..

[31] Neuman HB (2010). Stage IV breast cancer in the era of targeted therapy. Cancer.

[32] Nguyen DHA (2012). Can Locoregional Treatment of the Primary Tumor Improve Outcomes for Women With Stage IV Breast Cancer at Diagnosis?. Int. J. Radiat. Oncol..

[33] Pathy NB, Verkooijen HM, Taib NA, Hartman M, Yip CH (2011). Impact of breast surgery on survival in women presenting with metastatic breast cancer. Br. J. Surg..

[34] Pérez-Fidalgo JA (2011). Removal of primary tumor improves survival in metastatic breast cancer. Does timing of surgery influence outcomes?. The Breast.

[35] Pons-Tostivint E (2019). Survival Impact of Locoregional Treatment of the Primary Tumor in *De Novo* Metastatic Breast Cancers in a Large Multicentric Cohort Study: A Propensity Score-Matched Analysis. Ann. Surg. Oncol..

[36] Rapiti E (2006). Complete Excision of Primary Breast Tumor Improves Survival of Patients With Metastatic Breast Cancer at Diagnosis. J. Clin. Oncol..

[37] Rashaan ZM (2012). Surgery in metastatic breast cancer: Patients with a favorable profile seem to have the most benefit from surgery. Eur. J. Surg. Oncol..

[38] Ruiterkamp J (2011). Improved survival of patients with primary distant metastatic breast cancer in the period of 1995–2008. A nationwide population-based study in the Netherlands. Breast Cancer Res. Treat..

[39] Shibasaki S, Jotoku H, Watanabe K, Takahashi M (2011). Does primary tumor resection improve outcomes for patients with incurable advanced breast cancer?. Breast.

[40] Barinoff J (2017). Primary metastatic breast cancer in the era of targeted therapy – Prognostic impact and the role of breast tumour surgery. Eur. J. Cancer.

[41] Shien T (2009). Primary tumor resection improves the survival of younger patients with metastatic breast cancer. Oncol. Rep..

[42] Soran A (2018). Randomized Trial Comparing Resection of Primary Tumor with No Surgery in Stage IV Breast Cancer at Presentation: Protocol MF07-01. Ann. Surg. Oncol..

[43] Tan Y (2016). Hormone receptor status may impact the survival benefit of surgery in stage IV breast cancer: a population-based study. Oncotarget.

[44] Thomas A, Khan SA, Chrischilles EA, Schroeder MC (2016). Initial Surgery and Survival in Stage IV Breast Cancer in the United States, 1988-2011. JAMA Surg..

[45] Van Uden, D. J. P. *et al*. Metastatic behavior and overall survival according to breast cancer subtypes in stage IV inflammatory breast cancer. *Breast Cancer Res*. **21** (2019). 10.1186/s13058-019-1201-5 PMC6798447 31623649

[46] Wang, K. *et al*. Metastatic pattern discriminates survival benefit of primary surgery for *de novo* stage IV breast cancer: A real-world observational study. *Eur. J. Surg. Oncol*. **0** (2019). 10.1016/j.ejso.2019.02.013 30837102

[47] Wang W (2019). Impact of Locoregional Treatment on Prognosis of *de novo* Stage IV Breast Cancer: A Retrospective Long-Term Study of Chinese Population. Gynecol. Obstet. Invest..

[48] Warschkow R (2016). Improved Survival After Primary Tumor Surgery in Metastatic Breast Cancer. Ann. Surg..

[49] Weiss A (2018). Factors associated with improved outcomes for metastatic inflammatory breast cancer patients. Breast Cancer Res. Treat..

[50] Wu S-G (2017). The survival benefits of local surgery in stage IV breast cancer are not affected by breast cancer subtypes: a population-based analysis. Oncotarget.

[51] Bertaut A (2015). Stage IV breast cancer: a population-based study about prognostic factors according to HER2 and HR status. Eur. J. Cancer Care (Engl)..

[52] Xie Y (2017). Surgery of the primary tumor improves survival in women with stage IV breast cancer in Southwest China: A retrospective analysis. Medicine (Baltimore)..

[53] Xiong Z (2018). Could local surgery improve survival in *de novo* stage IV breast cancer?. BMC Cancer.

[54] Blanchard DK, Shetty PB, Hilsenbeck SG, Elledge RM (2008). Association of Surgery With Improved Survival in Stage IV Breast Cancer Patients. Ann. Surg..

[55] Chen P-Y, Cheng SH-C, Hung C-F, Yu B-L, Chen C-M (2013). Locoregional therapy in luminal-like and HER2-enriched patients with *de novo* stage IV breast cancer. Springerplus.

[56] Dawood S (2012). Identifying factors that impact survival among women with inflammatory breast cancer. Ann. Oncol..

[57] Desille-Gbaguidi H, Avigdor S, Body G, Ouldamer L (2019). Survival impact of primary site surgery on metastatic breast cancer patients at diagnosis. J. Gynecol. Obstet. Hum. Reprod..

[58] Dominici L (2011). Surgery of the primary tumor does not improve survival in stage IV breast cancer. Breast Cancer Res. Treat..

[59] Yang F, Xu J, Tang L, Guan X (2017). Breast cancer stem cell: the roles and therapeutic implications. Cellular and Molecular Life Sciences.

[60] Janssen LME, Ramsay EE, Logsdon CD, Overwijk WW (2017). The immune system in cancer metastasis: friend or foe?. J. Immunother. Cancer.

[61] Coffelt SB (2015). IL-17-producing γδ T cells and neutrophils conspire to promote breast cancer metastasis. Nature.

[62] Cardoso F (2016). ESO–ESMO International Consensus Guidelines for Advanced Breast Cancer (ABC 3). Ann. Oncol..

[63] Tohme S, Simmons RL, Tsung A (2017). Surgery for Cancer: A Trigger for Metastases. Cancer Res..

[64] Castaño Z (2018). IL-1β inflammatory response driven by primary breast cancer prevents metastasis-initiating cell colonization. Nat. Cell Biol..

[65] Cady B, Nathan NR, Michaelson JS, Golshan M, Smith BL (2008). Matched Pair Analyses of Stage IV Breast Cancer with or Without Resection of Primary Breast Site. Ann. Surg. Oncol..

[66] Cristofanilli M (2019). The clinical use of circulating tumor cells (CTCs) enumeration for staging of metastatic breast cancer (MBC): International expert consensus paper. Crit. Rev. Oncol. Hematol..

